# Enhancing nutritional value and flavor profiles of whey powder through fungal fermentation with *Aspergillus oryzae* and *Neurospora intermedia*

**DOI:** 10.1177/10820132251368707

**Published:** 2025-08-17

**Authors:** Burcu Kaya, Onur Güneşer, Mohammad J Taherzadeh, Yonca Karagül Yüceer, Taner Sar

**Affiliations:** 1 Swedish Centre for Resource Recovery, 52950 University of Borås, Borås, Sweden; 2 Department of Food Engineering, Çanakkale Onsekiz Mart University, Çanakkale, Türkiye; 3 Engineering Faculty, Department of Food Engineering, 175652 Uşak University, Uşak, Türkiye

**Keywords:** Whey, fungal fermentation, antioxidant activity, alternative food source

## Abstract

Investigation of the nutritional properties, biological activities, volatile compounds and sensory properties of fungal biomass and supernatants obtained from cheese whey powder fermented with *Aspergillus oryzae* and *Neurospora intermedia* was aimed in this study. The biomass produced by *A. oryzae* exhibited higher total lipid (118.54 g/kg) and total essential amino acid (62.05 g/kg) contents improve in comparison to *N. intermedia*. In contrast, the *N. intermedia* biomass showed superior bioactive properties, with the highest levels of total phenolics (4.72 mg gallic acid/g dry basis), total flavonoids, (23.85 mg quercetin/dry basis), and antioxidant activities (221.49 mg Trolox/g dry basis). Furthermore, the *A. oryzae* biomass derived from whey powder significantly enhanced the concentration of 1-octen-3-ol from 15.64 to 129.35 µg/kg, indicating its potential for improving the flavor profiles of food products with a natural mushroom-like aroma. Whey powder fermented with *A. oryzae* and *N. intermedia* contained significant amounts of calcium, sodium, and magnesium. The dominant mineral in the supernatant was Mg (7.40–7.90 mg/L) and a distinct fruity aroma was observed especially in the *N. intermedia* supernatant. These findings highlight the potential of fungal fermentation to convert dairy industry byproducts into nutrient-dense, flavor-enhancing alternatives.

## Introduction

Fungi are excellent sources of protein (40%–50%, crude), fiber (chitin-glucan matrix), and a wide range of vitamins, micronutrients, and antioxidants (Rousta et al., 2022). Additionally, due to their rich content of essential amino acids and fatty acids (including gamma-linolenic acid, omega-6, and omega-3 fatty acids), fungi are regarded as a promising alternative food source for human nutrition (Ahmad et al., 2022; Vongsangna and Nielsen, 2013).

The primary challenge in microbial biomass production is sourcing cost-effective, nutrient-rich cultivation media, which can potentially be addressed by utilizing organic-rich industrial waste or byproducts. This approach not only reduces production costs but also minimizes the environmental impact of waste disposal (Karimi et al., 2019). Previous studies have demonstrated that fungi can grow on various substrates including potato protein liquor, bread leftover, oat husk, and pea byproduct (Rousta et al., 2024).

Whey is a clear, yellowish to greenish liquid separated from curd produced by rennet or acid coagulation of milk (Guo and Wang, 2019). The global annual cheese production is estimated at 20.5 million tons, generating approximately 185 million tons of whey. However, only 55.5 million tons are processed into cheese whey powder each year (Domínguez-Niño et al., 2018). The primary components of whey are lactose (45–50 g/L), soluble proteins (6–8 g/L), lipids (4–5 g/L), and mineral salts (NaCl, KCl, and calcium). Additionally, whey contains lactic acid (0.5 g/L), citric acid, urea, uric acid, and B-group vitamins (Carvalho et al., 2013). Due to its rich nutritional composition, cheese whey serves as a promising substrate for microbial fermentation, offering significant potential for conversion into value-added products, for example, bioethanol, enzymes, beverage, biopolymer, or sing cell protein (Kaya et al., 2024; Sar et al., 2022a). Furthermore, its acidic pH supports the growth of filamentous fungal biomass (Carvalho et al., 2013). Mahboubi et al. (2017) reported that biomass concentrations between 7 and 16 g/L can be achieved when cultivating *Aspergillus oryzae* and *Neurospora intermedia* in cheese whey. Although this and similar studies demonstrate the potential of using dairy industry byproducts for fungal biomass production, biomass characterization has not been performed.

Whey-based beverages, first produced on an industrial scale in the 1970s, now come in a variety of forms, including fermented and nonfermented, alcoholic, diet, and high-protein options (Zotta et al., 2020). Fermented whey beverages are typically produced using lactic acid bacteria, and yeast strains (Zotta et al., 2020). Despite these developments, there is a notable absence of whey-based beverages produced through the fermentation of filamentous fungi in the existing literature.

*Aspergillus oryzae* is predominantly known as the fungus responsible for the fermentation of koji, a traditional product mainly found in Asia (Lee et al., 2021; Zhao et al., 2020). It secretes various enzymes such as amylase, protease, and peptidase, producing beneficial metabolites like fatty acids and amino acids from the substrate (Lee et al., 2021). *N. intermedia*, the key yeast of oncom merah (red oncom), a traditional product in Java, Indonesia (Bulkan et al., 2022; Rekdal et al., 2023). Both microorganisms have been approved as “Generally Recognized as Safe” (GRAS) for food use by the Food and Agriculture Organization of the United Nations (FAO) (Gamarra-Castillo et al., 2022; Osadolor et al., 2017).

The aim of this study was to identify the nutritional characteristics of fungal biomasses produced from whey and to investigate their potential applications in human nutrition. Additionally, the study explored to elucidate the differences in nutritional value between the biomasses of *A. oryzae* and *N. intermedia*. To achieve this, the biomasses have been characterized in terms of various properties including amino acid and fatty acid profiles, mineral contents, biological activities (total phenolic, total flavonoid, and antioxidant activity), volatile compounds, and sensory attributes.

## Materials and methods

### Fungal biomass

The biomass of *A. oryzae* var. *oryzae* CBS 819.72 (Centraalnureau Voor Schimmel cultures, Utrecht, and The Netherlands) and *N. intermedia*, CBS 131.92, along with their whey powder culture supernatants, was cultivated in a bubble column bioreactor using whey powder and detailed in the study conducted by Kaya et al. (2024). The bubble column reactors (Belach Bioteknik, Stockholm, Sweden) were filled with 3 L of substrate that contained 3% (w/v) of cheese whey powder. After adjusting the initial pH to 5.0 using 2 M H₂SO₄, sterilization was performed at 121 °C for 20 minutes. The medium was inoculated with 20 mL/L of fungal spore suspension and incubated at 35 °C for 48 hours (with aeration rate of 1.0 volume of air per volume of medium per minute). Prior to analysis, the harvested biomass was freeze-dried at 0.05 bar and −50 °C.

### Total lipids contents and fatty acids compositional analysis

Total lipid contents were measured using a Soxtec manual apparatus (ST243 SoxtecTM, FOSS Analytical Co., Ltd, Suzhou, China) using petroleum ether as a solvent. For lipid extraction, fungal biomass (2 g) was mixed with 30 mL of chloroform:methanol (2:1, v/v), and then the mixture was incubated in a shaking incubator at 200 rpm. It was subjected to sonication for 5 minutes every 30 minutes. Then the mixture was centrifugated and the solvent was evaporated using a rotary evaporator at 40 °C. And 5 mL of hexane and 0.25 mL of KOH (2 M) were used for fatty acid methyl esters (Tuncel et al., 2017). Then, the solvent phase was evaporated under nitrogen and concentrated. The fatty acids methyl esters were analyzed through an Agilent 6890N gas chromatograph (Agilent Technologies, USA) equipped with a flame ionization detector and an HP-88 (100 m × 250 µm × 0.25 µm) column (Agilent Technologies, USA). The carrier gas was helium at a flow rate of 2 mL/min. The temperature program was as follows: hold at initial temperature of 120 °C for 1 min; increase to 175 °C at a rate of 10 °C/min, increase to 210 °C at a rate of 5 °C/min; hold at 210 °C for 5 minutes; increase to 230 °C at a rate of 5 °C/min; hold at 230 °C for 5 minutes. The sample injection volume was 1 μL. The fatty acid composition was determined using a standard mixture, Supelco^®^ 37-component (Sigma-Aldrich, USA), based on retention time. The fatty acid concentrations of the fungal biomass samples were determined using peak normalization technique and expressed as percentage.

### Determination of mineral contents

Minerals including calcium (Ca), potassium (K), sodium (Na), iron (Fe), magnesium (Mg), zinc (Zn), copper (Cu), manganese (Mn), and chromium (Cr) were determined through Microwave Plasma-Atomic Emission Spectroscopy (MP-AES 4200, Agilent Technologies, Santa Clara, CA, USA) according to Sar et al. (2024).

### Determination of total amino acid composition

Total amino acid profiles of the fungal biomass samples were determined according to the method of Henderson et al. (2000) using the Agilent 1260 Infinity HPLC System (Agilent Technologies Inc., Folsom, CA, USA) consisting of Infinity 1260 Autosampler, 1260 Multiple Wavelength UV detector, and ZORBAX Eclipse-AAA (4.6 × 150 mm, 3.5 μ PN 963400-902). OPA (o-phthaldialdehyde, 10 mg/mL) and FMOC (fluorenylmethyloxycarbonyl chloride, 25 mg/mL) were used for amino acid derivatization with online derivatization technique performed by infinity 1260 Autosampler; 40 mM Na_2_HPO_4_ at pH 7.8 (Mobil phase A) and acetonitrile:methanol:water 45:45:10 (Mobil phase B) were used for chromatographic separation. Mobil phase condition and flow rate were: 0–1.9 minutes, solvent A 100%, and 1.9 to 18 minutes, solvent B from 0 to 57%; 18 to 18.6 minutes, solvent B increased to 100%; 18.6 to 22.3 minutes, solvent B was kept at 100%; 22.3 to 26, solvent B decreased from 100% to 0%. Flow rate was 2 mL/min. The column temperature was 40 °C. The external standard curve method with 17 amino acid mix (Sigma-Aldrich, No 79248, USA) standard was used for quantification of amino acids (*R*^2^ > 0.99). Content of amino acids in fungal biomass were expressed as mg/kg.

### Extraction of bioactive compounds

Bioactive compounds were extracted from the fungal biomass by using 80% ethanol. In briefly, 1 g of fungal biomass was mixed with 30 mL of ethanol and the mixture was placed in a shaker for 1 hour at 25 °C in order to homogenize (Corrêa et al., 2015). Then, the mixture was centrifuged (5.000 rpm, 2 minutes) and the supernatant was separated. The extraction procedure for 1 g sample was performed in duplicate. The whey powder culture supernatants were used without undergoing an extraction process.

### Determination of total phenolic contents

Total phenolic contents of the fungal biomass samples were determined using the Folin–Ciocalteu method (Çelebi Uzkuç et al., 2023; Singleton and Rossi, 1965). The sample extract (1 mL) was mixed with 5 mL of Folin–Ciocalteu's reagent, and 4 mL of 7.5% saturated sodium carbonate solution in a glass tube. The content of tube was mixed using a shaker and then incubated in darkness at room temperature for 1 hour. Afterward, the absorbance of the sample was read using ultraviolet (UV) spectrophotometer (UV-1800, Shimadzu, Japan) at 765 nm. A standard curve was generated by utilizing gallic acid in concentrations ranging from 40 to 320 mg/mL as a reference standard. The results were expressed as milligrams of gallic acid equivalent per gram of dry basis of fungal biomass (mg GAE/g dry basis). For the supernatant, it is expressed as milligrams of gallic acid equivalent per milliliter of supernatant (mg GAE/mL supernatant).

### Determination of total flavonoids contents

Flavonoid contents of the fungal biomass samples were determined using the method mentioned by Gong et al. (2018). The sample extract (0.1 mL) was mixed with 4.0 mL of distilled water and 0.3 mL of a 5% (w/v) NaNO_2_, then allowed to stand for 5 minutes. A quantity of 0.3 mL of AlCl was introduced into the mixtures, and the solution was allowed to stand for 1 minutes. Finally, 2 mL of NaOH and 2.4 mL of distilled water were added, and the absorbance was immediately measured using UV spectrophotometer (UV-1800, Shimadzu, Japan) at 510 nm. A quercetin line was used to quantify the total flavonoid content of the samples. Results are expressed as milligrams of quercetin per gram of dry matter (mg QE/g dry basis). For the supernatant, it is expressed as milligrams of quercetin equivalent per milliliter of supernatant (mg QE/mL supernatant).

### Determination of Trolox equivalent antioxidant capacity

ABTS (2,2′-azinobis(3-ethylbenzothiazoline-6-sulfonic acid)) radical scavenging method was used to measure antioxidant activity of the samples (Re et al., 1999). A stable ABTS radical solution was prepared by combining 0.0384 g of ABTS radical with a 12.25 mM potassium persulfate solution (2.0 mL) and completing the volume to 10 mL with distilled water in a volumetric flask. The reaction mixture was allowed to stand for 12 to 16 hours until the absorbance stabilized. The ABTS solution was diluted with phosphate buffer solution (pH 7.4) to produce an absorbance of 0.700 (±0.02) at 734 nm before the analysis. A sample extract was mixed with PBS (phosphate buffered saline)-diluted ABTS solution (1 mL), and after a 6-minute incubation at room temperature in the dark, absorbance was measured at 734 nm (UV-1800, Shimadzu, Japan). The results of antioxidant activity were expressed as milligrams of Trolox equivalent per kg of dry matter of fungal biomass (mg Trolox/kg dry basis). For the supernatant, it is expressed as milligrams of Trolox equivalent per milliliter of supernatant (mg Trolox/mL supernatant).

### Extraction, identification, and quantification of volatile compounds

The volatile compounds of fungal biomass were extracted using solid phase microextraction (SPME) according to Güneşer et al. (2016) with some modifications. A 0.2 g of freeze-dried fungal biomass was mixed with 5 mL of a saturated NaCl in a 40 mL amber colored screw top vial with a PTFE (Polytetrafluoroethylene)/silicon septum hole cap (Supelco, Bellafonte, USA). The vial was placed in a waterbath (GFL, Grossburgwedel, Germany) at 40 °C for 20 minutes. Headspace sampling was performed using a manual SPME holder (2 cm to 50/30 μm DVB/Carboxen/PDMS, Supelco, Bellafonte) held in the headspace of the vial at a depth of 2 cm for 20 minutes at 40 °C. The flavor compounds of fungal biomass samples were identified using gas chromatography–mass spectrometry (GC-MS). The GC-MS system consisted of HP-INNOWax column (60 m × 0.25 mm ID × 0.25 μm film thickness, J&W Scientific, Folsom, CA), and MS 6890 GC (Agilent Technologies, Wilmington, DE, USA). The oven temperature was programmed as 40°C to 250 °C, at 5 °C/min; initial hold time of 40 °C and 1 minute, final hold time 250 °C and 20 minute. The capillary direct interface temperature was 250 °C; carrier gas, helium adjusted to constant flow of 1.2/min; ionization energy, 70 eV; mass range, 35 to 350 amu; scan rate 4.45 scans/s. The identification of volatile compounds was accomplished by a mixture of internal standards (using 2-methyl-pentanoic acid and 2-methyl-3-heptanone for acidic and neutral-basic compounds, respectively), retention index (RI), and comparison with the mass spectral data libraries.

### Descriptive sensory analysis

Sensory evaluation was conducted with six panelists (1 male and 5 female), aged between 24 and 55 years, who had prior experience in evaluating various products using the *Spectrum*^TM^ procedure. Descriptive terms and changes in aroma profiles of both fungal biomass and supernatants were assessed by the panelists. A sample of dried fungal biomass (1 g) was presented to the panelists in containers (30 mL). The supernatant samples were distributed into capped containers in 5 mL aliquots and presented to the panelists. Panelists did not swallow the samples then spat them out. Room temperature water was provided for palate cleansing between samples. Standard solutions were provided for umami (0.5% monosodium glutamate), bitter (0.05% caffeine), sweet (0.2% sucrose), sour (0.05% citric acid), and salty (0.2% sodium chloride) tastes. In addition, cheese whey powder, grass, hazelnut, pepper, lemon, mushroom, resin, rose water, almond, and cucumber, presented in 30 mL containers, were used as reference materials. Fifteen-point product-specific scale anchored to the left with “not” and to the right with “very” was used for evaluation of the samples (Meilgaard et al., 1999).

### Statistical analysis

Data were presented as means ± standard error of the mean based on duplicated experiments. The statistical significance mineral content, total phenolic content, total flavonoid content, and antioxidant activity was analyzed by one-way analysis of variance (ANOVA) (*p* ≤ 0.05) using SPSS 23.0 (SPSS Inc., Chicago, IL, USA). The differences between the samples, and the pairwise comparisons were made using Duncan's multiple range test (Sheskin, 2004). The statistical significance fatty acids were analyzed by an independent samples t test (*p* ≤ 0.05). The relationship between volatile compounds and sensory attributes was visualized using Principal Component Analysis (PCA) in the XLSTAT (free trial version). Heatmaps of total amino acid content of the fungal biomass were established by using GraphPad Prism 9 Software (free trial version, GraphPad Sofware, LLC, San Diego, California USA). Experimental results were means of duplicated analysis.

## Results and discussion

In this study, the nutritional properties of biomasses produced from whey were determined, and their potential use in human nutrition was discussed. In this context, the biomasses were characterized in terms of various properties, including amino acid and fatty acid profiles, mineral content, biological activities (total phenolics, total flavonoids, and antioxidant activity), volatile compounds, and sensory attributes.

### Nutritional properties of fungal biomass

#### Amino acid profiles

**Figure 1.**
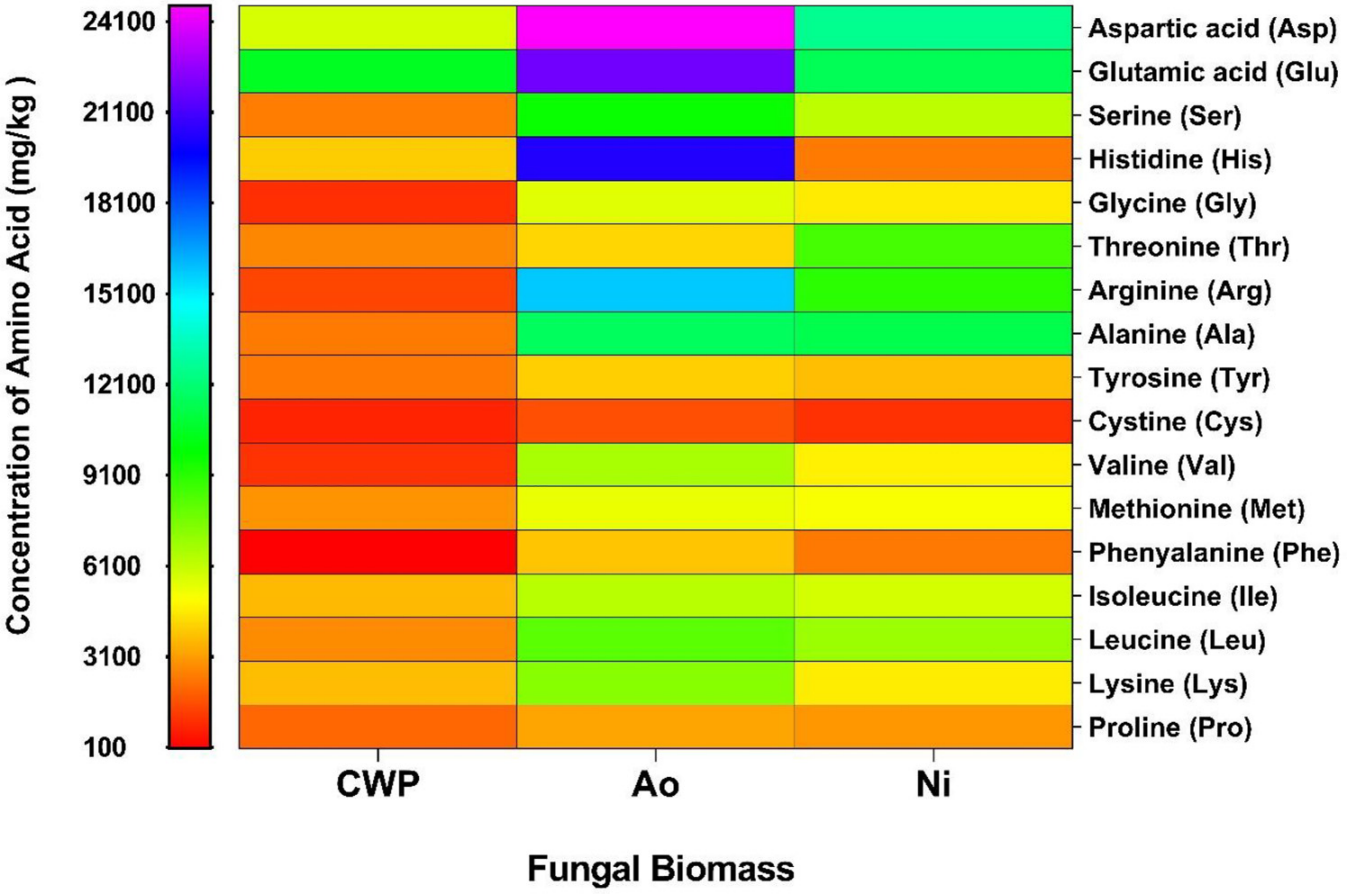
Heatmap of the total amino acid contents in whey powder, *Aspergillus oryzae* biomass, and *Neurospora intermedia* biomass.

Heatmaps graph illustrating the amino acid profiles of the fungal biomass and whey powder were shown in Figure 1. Whey powder exhibited the lowest amino acid content, particularly in glycine (Gly), tyrosine (Tyr), methionine (Met), leucine (Leu), lysine (Lys), and proline (Pro). Significant differences were observed in the total amino acid content of fungal biomasses when compared to whey powder and across the fungal species used. Although the biomass of *A. oryzae* and *N. intermedia* displayed similar amino acid properties, the *A. oryzae* biomass contained higher aspartic acid (Asp), glutamic acid (Glu), histidine (His), arginine (Arg), alanine (Ala), valine (Val), leucine (Leu), and lysine (Lys) contents compared to *N. intermedia*. Similarly, Rousta et al. (2022) stated that the amino acid profile of *A. oryzae* biomass derived from oat flour was dominated by Asp, Glu, Ala, Val, Leu, and Lys. The differences in Asp, Glu, His, Thr, Arg, Val, Phe, and Lys concentrations can be attributed to differences in fungal species. The essential amino acid content of whey powder (20.76 g/kg) was increased to 62.05 and 40.42 g/kg when fermented with *A. oryzae* and *N. intermedia*, respectively. Comparable increases in essential amino acid content had been observed following the fermentation of substrates such as oat flour, potato protein liquor, vinasse, cheese whey, and orange molasses by various fungal species (Ibarruri and Hernández, 2019; Karimi et al., 2019; Rousta et al., 2022; Sar et al., 2022b).

Histidine, the primary essential amino acid in whey powder, exhibited significant changes in concentration after fermentation: its level increased by 421.46% in *A. oryzae* biomass, while it decreased by 38.57% in *N. intermedia* biomass (Figure 1). The World Health Organization estimated the daily histidine requirement for adults to be between 8 and 12 mg per kg of body weight (FAO/WHO/UNU, 1985; Thalacker-Mercer and Gheller, 2020). It has been suggested that histidine may help reduce long-term mental stress and fatigue, as well as mitigate the negative effects of certain types of cancer (Cynober et al., 2020). In the present study, the histidine content in *A. oryzae* biomass produced in whey powder exceeds the daily intake level, while 1 g of *N. intermedia* biomass meets 20.33% to 30.5% of this requirement. Furthermore, fungal fermentation significantly increased the phenylalanine content of whey powder, with levels rising by 61.49 times in *A. oryzae* biomass (3.85 mg/g) and by 37.87 times in *N. intermedia* biomass (2.37 mg/g). Phenylalanine is an essential amino acid crucial for the proper functioning of the central nervous system (Akram et al., 2020).

#### Lipid contents and fatty acid compositions

Whey powder contains a high concentration of lactose (86.32%, w/w) and a low lipid content (0.5%, w/w) (Kaya et al., 2024). The biomasses of *A. oryzae* and *N. intermedia* cultivated from whey powder were found to contain 118.54 and 25.26 g/kg lipids, respectively. The results indicate that lipid content as well as protein content of whey powder can be enhanced through fungal bioconversion. It has been shown in literature that the lipid content of fungal biomass cultivated on various substrates can vary between 4% to 22% (Karimi et al., 2021; Rousta et al., 2022). High glucose concentration or altered carbon-to-nitrogen (C/N) ratios in the fermentation medium can trigger the fatty acid synthesis pathway in fungi, resulting in increased fat content (Rousta et al., 2022). The significant accumulation of fat is primarily stored within the cells of *A. oryzae*, particularly in its filamentous hyphae structure (Mahboubi et al., 2017). This phenomenon may explain the higher fat content observed in *A. oryzae* compared to the biomass of *N. intermedia*.

The fatty acid composition of the fungal biomasses was shown in Table 1. In both fungal biomasses, the predominant fatty acid group was saturated fatty acids (SFA, 38.85%–41.85%), followed by monounsaturated fatty acids (MUFA, 31.32%–39.47%) and then polyunsaturated fatty acids (PUFA, 19.35–29.82%). The most abundant fatty acids identified in the fermented biomasses were linoleic acid (C18:2 cis), oleic acid (C18:1 cis), and palmitic acid (C16:0). This pattern aligns with a previous study conducted by fermentation of olive oil using *A. oryzae* (Nazir et al., 2022). Additionally, the fermentation of oat flour with *A. oryzae* and bread with *N. intermedia* yielded similar profiles of primary fatty acids in the resulting fungal biomass (Gmoser et al., 2020; Rousta et al., 2022). In this research, the highest concentration of palmitic acid (29.79%) was detected in *A. oryzae* biomass, while oleic acid (33.36%) was most prevalent in *N. intermedia* biomass (*p* ≤ 0.05).

**Table 1.** Fatty acid profiles of fungal biomass samples.

		Fatty acid contents (% of total FA)		
Abbreviation	Fatty acid	*Aspergillus oryzae*	*Neurospora intermedia*	p-Value
C4:0	Butyric acid	0.29 ± 0.02	2.09 ± 0.31	0.001*
C6:0	Caproic acid	0.04 ± 0.00	nd	
C8:0	Caprylic acid	0.05 ± 0.00	2.01 ± 0.21	0.000*
C12:0	Lauric acid	0.08 ± 0.00	2.29 ± 0.18	0.000*
C14:0	Myristic acid	1.87 ± 0.08	7.19 ± 0.14	0.000*
C14:1	Myristoleic acid	0.16 ± 0.01	0.91 ± 0.02	0.000*
C15:0	Pentadecanoic acid	0.94 ± 0.04	0.73 ± 0.02	0.002*
C16:0	Palmitic acid	29.79 ± 1.16	19.33 ± 5.07	0.025*
C16:1	Palmitoleic acid	2.62 ± 0.23	2.74 ± 0.32	0.638
C17:0	Margaric acid	0.59 ± 0.16	nd	
C17:1	Heptadecanoic acid	0.77 ± 0.01	1.28 ± 0.11	0.002*
C18:0	Stearic acid	4.75 ± 1.73	6.48 ± 0.61	0.176
C18:1 cis	Oleic acid (n9)	27.77 ± 0.15	33.36 ± 3.61	0.056*
C18:2 cis	Linoleic acid (n6)	28.96 ± 0.42	18.12 ± 1.99	0.001*
C18:3n6	Gamma linoleic acid	0.58 ± 0.10	nd	
C20:0	Arachidic acid	0.46 ± 0.02	0.97 ± 0.06	0.000*
C20:5	Eicosapentaenoic acid	nd	0.65 ± 0.21	
C22:1	Erucic acid	nd	1.18 ± 0.21	
C22:2	Cis-13, 16-docosadienoic acid	0.28 ± 0.06	0.19 ± 0.09	0.278
C22:6	Docosahexaenoic acid	nd	0.39 ± 0.08	

	Total FA	100	100	
	Total SFA	38.85 ± 0.61	41.85 ± 5.41	0.518
	Total MUFA	31.32 ± 0.35	39.47 ± 3.63	0.018*
	Total PUFA	29.82 ± 0.24	19.35 ± 1.77	0.001*

FA: fatty acids; MUFA: monounsaturated fatty acids; nd: not detected; PUFA: polyunsaturated fatty acids; SFA: saturated fatty acids.

Comparing the fatty acids profile of *A. oryzae* biomass with *N. intermedia* biomass, it was found that *A. oryzae* can produce gamma linoleic acid, margaric acid and caproic acid, whereas *N. intermedia* cannot. Conversely, *N. intermedia* can produce docosahexaenoic acid (DHA), erucic acid, and eicosapentaenoic acid (EPA), which are not synthesized by *A. oryzae*. This finding is consistent with Pinasthika et al. (2018), who also reported that *A. oryzae* is unable to synthesize DHA and EPA. According to Arcanggi et al. (2018), optimal production of unsaturated fatty acids in *A. oryzae* requires an appropriate carbon concentration and agitation rate. Additionally, the authors emphasized that excessively high agitation rates may induce shear stress in *A. oryzae*, which could explain the absence of DHA and EPA in its biomass. Thus, the inability to detect DHA and EPA in the biomass of *A. oryzae* could be attributed to this phenomenon.

In the current study, the levels of caprylic acid (2.01%), lauric acid (2.29%), myristic acid (7.19%), and stearic acid (6.48%) in *N. intermedia* biomass was higher than in *A. oryzae* biomass. Dayrit (2015) emphasized that these fatty acids exhibit antimicrobial properties against different groups of microorganisms (*Staphylococcus aureus*, *Pseudomonas aeruginosa*, *Streptococcus* group A, *Candida albicans,* Vesicular stomatitis virus), with lauric acid being particularly noted as the most active among saturated fatty acids.

Omega-3 (n-3), -6 (n-6), and -9 (n-9) fatty acids are unsaturated fatty acids that offer various health benefits for human (Farag and Gad, 2022). Omega-3 is crucial for reducing cardiovascular diseases and neurological disorders, omega-6 supports brain development, and omega-9 exhibits anti-inflammatory and anticancer properties (Farag and Gad, 2022; Patel et al., 2022). In our study, both biomasses were significant sources of omega-6 and omega-9 fatty acids. Furthermore, *N. intermedia* contains erucic acid (1.18%), a member of the omega-9 family.

A ratio below 0.45 between unsaturated and saturated fatty acids (FAU/FAS) in any given food is not recommended for health due to its potential association with cardiovascular diseases (Denardi-Souza et al., 2018). In this study, the FAU/FAS ratios were found to be 1.57 for *A. oryzae* and 1.40 for *N. intermedia*. Given these ratios and the overall fatty acid composition, the biomasses produced in our study show potential applications in human nutrition. Notably, the diversity in the composition of *N. intermedia* biomass could make it particularly appealing for food applications.

### Mineral contents

The ash content in whey powder was determined as 72.11 g/kg (Kaya et al., 2024). The characteristics of milk and seasonal variations can significantly influence the mineral content of whey. For instance, Franceschi et al. (2023) reported that the average phosphorus (P) and magnesium (Mg) contents of whey were higher during autumn and winter, and lower during summer. Some studies have reported that whey may contain elements such as calcium (Ca), potassium (K), sodium (Na), iron (Fe), magnesium (Mg), zinc (Zn), copper (Cu), manganese (Mn), and chromium (Cr) (González-Weller et al., 2023; Mehra et al., 2022). The results highlight that minerals like Ca, K, and Na are present in notably higher concentrations, suggesting their fundamental role in whey powder (Table 2). This is further supported by additional research on whey (González-Weller et al., 2023; Mehra et al., 2022).

**Table 2.** Mineral levels of the whey powder, biomass, and supernatant samples.

M	Whey powder	Biomass		Supernatant		
		*Aspergillus oryzae*	*Neurospora intermedia*	Initial	*A. oryzae*	*N. intermedia*
Ca^1^	2.40 ± 0.21c	3.48 ± 0.0 0b	5.24 ± 0.02a	0.10 ± 0.01b	0.10 ± 0.01b	0.13 ± 0.01a
K^1^	8.60 ± 0.10b	14.92 ± 0.42a	5.67 ± 0.01c	0.20 ± 0.01a	0.08 ± 0.00b	0.08 ± 0.01b
Na^1^	2.28 ± 0.13c	6.55 ± 0.29b	11.83 ± 0.02a	0.39 ± 0.01a	0.26 ± 0.01b	0.40 ± 0.04a
Fe^2^	313.36 ± 36.22a	228.93 ± 12.63b	3.33 ± 0.61c	0.10 ± 0.05b	0.09 ± 0.02b	0.58 ± 0.09a
Mg^2^	441.87 ± 6.20c	964.14 ± 33.53a	599.73 ± 0.78b	7.90 ± 0.07	7.50 ± 0.08	7.40 ± 0.70
Zn^2^	44.16 ± 2.56a	40.57 ± 1.54a	13.70 ± 0.88b	0.08 ± 0.04	0.03 ± 0.00	0.03 ± 0.01
Cu^2^	7.85 ± 0.33b	11.91 ± 1.08a	nd	0.01 ± 0.00a	0.01 ± 0.00 ab	nd
Mn^2^	7.38 ± 0.49b	10.21 ± 0.80a	1.45 ± 0.01c	0.09 ± 0.09	0.07 ± 0.00	0.04 ± 0.00
Cr^2^	118.69 ± 12.81a	71.32 ± 2.01b	nd	0.02 ± 0.00c	0.32 ± 0.04b	0.54 ± 0.02a

Initial: 3% (w/v) unfermented cheese whey.

Statistical analysis has been performed separately for the biomass and supernatant sample groups. Different letters indicate statistical differences among the samples (p ≤ 0.05).

1 Unit of Ca, K, and Na: g/kg dry basis biomass, g/L supernatant.

2 Unit of Fe, Mg, Zn, Cu, Mn, and Cr: mg/kg dry basis biomass, mg/L supernatant.

M: mineral type; nd: not detected.

The production of fungal biomass by *A. oryzae* and *N. intermedia* resulted in a significant increase in the calcium (Ca), sodium (Na), and magnesium (Mg content of whey powder ; *p* ≤ 0.05). Similarly, studies by Karimi et al. (2019) and Rousta et al. (2022) reported increased mineral content, specifically calcium, sodium, and magnesium, in vinasse and oat flour following fermentation with variety of fungal species. Calcium (Ca), the primary component of bone, is essential for achieving optimal bone density during youth and maintaining it throughout life (Areco et al., 2015). The recommended daily intake of calcium is 900 mg for adults, and 1200 mg for adolescents and elder people (Guéguen and Pointillart, 2000). The fungal biomass produced in this study provides approximately 38% to 58% of the daily calcium requirement for adults and 29 to 43% for adolescents and older adults per 100 g. The sodium (Na) requirement of males and females ranges from 1.2 to 1.5 g per day from food sources (Quintaes and Diez-Garcia, 2015). Notably, 100 g of *A. oryzae* biomass provide 55% of the daily sodium needs, while *N. intermedia* biomass meets 100% of the daily requirement. The primary source of magnesium (Mg) for humans is animal-based foods, and Mg levels essential for human cellular metabolism (Quintaes and Diez-Garcia, 2015). The Mg contents of *A. oryzae* and *N. intermedia* biomasses were found to be 964.14 and 599.73 mg/kg, respectively.

The levels of potassium (K), copper (Cu), and manganese (Mn) in *A. oryzae* biomass were significantly higher than those in whey powder (*p* ≤ 0.05). On the other hand, the levels of iron (Fe) and zinc (Zn) in both fungal biomasses were lower compared to whey powder. In addition, the Cr level in *A. oryzae* biomass was also found to be significantly lower than whey powder (*p* ≤ 0.05), and the minerals Cu and Cr were not detected in *N. intermedia* biomass. Compared to Quorn, which is used as a mycoprotein source, biomass of *A. oryzae* and *N. intermedia* is rich in macronutrients but exhibits low levels of certain micronutrients such as magnesium (Mg), zinc (Zn), copper (Cu), and manganese (Mn) (Finnigan et al., 2016). However, in the present study, *A. oryzae* biomass produced from whey powder was found to contain higher levels of iron (Fe), zinc (Zn), and copper (Cu) compared to the koji samples produced from wheat, rice, brown rice, and soybeans (Nemoto et al., 2020).

In the supernatants of *A. oryzae* and *N. intermedia*, the level of chromium (Cr) has significantly increased compared to the initial levels (*p* ≤ 0.05). Additionally, there were significant increases in the levels of calcium (Ca) and iron (Fe) in the supernatants of *N. intermedia* (*p* ≤ 0.05). However, for the supernatants of both fungal strains, the levels of potassium (K), magnesium (Mg), zinc (Zn), and manganese (Mn) were found to be lower than the initial levels. In athletes engaged in intense exercise, dehydration can occur due to the inability to maintain fluid balance, leading to the loss of minerals such as sodium (Na), potassium (K), calcium (Ca), and magnesium (Mg) (Çağiran et al., 2023). Considering the notable magnesium content, it is suggested that supernatants can be used as a potential sports drink.

### Total phenolic contents, total flavonoid contents, and antioxidant activities

The phenolic content of whey powder was determined as 2.028 mg GAE/g dry substrate and increased by 94.67% and 133.13% following cultivation with *A. oryzae* and *N. intermedia*, respectively (*p* ≤ 0.05; Table 3). However, the total phenolic contents of supernatants *A. oryzae* and *N. intermedia* were lower than the whey powder. The highest total flavonoid content was determined in the *N. intermedia* fungal biomass (23.850 mg quercetin/g dry basis), which was statistically significant (*p* ≤ 0.05; Table 3). Meanwhile, the total flavonoid content increased in fungal biomasses compared to whey powder (*p* ≤ 0.05). No statistically significant differences were determined in the flavonoid contents of the fermented whey by *A. oryzae* and *N. intermedia*. The antioxidant activity levels for whey powder, *A. oryzae*, and *N. intermedia* biomasses were 104.34, 175.56, and 221.49 g Trolox/kg dry basis, respectively (Table 3). The antioxidant activity of whey was attributed to the bioactive peptides (Hernández-Ledesma et al., 2011). In addition, results of antioxidant activity indicated that the antioxidant activity of whey powder could be significantly enhanced through fungal bioconversion (*p* ≤ 0.05).

**Table 3.** Total phenolic contents, total flavonoid contents, and antioxidant activities of the samples.

Samples	Total phenolic content^1^	Total flavonoid content^2^	Antioxidant activity^3^
WP	2.028 ± 0.00c	0.600 ± 0.00c	104.34 ± 24.46c
Ao	3.948 ± 0.00b	10.500 ± 0.00b	175.56 ± 13.39b
AoS	0.073 ± 0.02d	0.089 ± 0.03d	0.038 ± 0.00d
Ni	4.728 ± 0.51a	23.850 ± 0.42a	221.49 ± 1.77a
NiS	0.090 ± 0.00d	0.140 ± 0.00d	0.033 ± 0.00d

Different letters indicate statistical differences among the samples (p ≤ 0.05).

1 Unit of total phenolic content: mg GAE/g dry basis, mg GAE/mL supernatant.

2 Unit of total flavonoid content: mg QE/g dry basis, mg QE/mL supernatant.

3 Unit of antioxidant activity: mg Trolox/g dry basis, mg Trolox/mL supernatant.

Ao: dry biomass of *Aspergillus oryzae*; AoS: supernatant of *Aspergillus oryzae*; Ni: dry biomass of *Neusrospora intermedia*; NiS: supernatant of *Neurospora intermedia*; WP: whey powder.

*A. oryzae* cultivation has been shown to increase total phenolic contents and antioxidant activities in wheat bran and sorghum (Espitia-Hernández et al., 2022; Yin et al., 2018), which align with our results. Additionally, Stodolak et al. (2020) reported that fermentation through *A. oryzae* and *N. intermedia* significantly increases antioxidant capacities of flaxseed oil cake. Researchers suggest that hydrolytic enzymes (e.g. β-glucosidase) produced during fermentation with the *A. oryzae* strain may increase the concentration of free phenolic compounds in rice bran (Rashid et al., 2018; Shin et al., 2019). Aryal et al. (2019) found a strong correlation between the total phenolic content, total flavonoid content, and antioxidant activity. Cai et al. (2012) reported that the amounts and/or activities of enzymes produced by fungi can vary depending on the strain, affecting the total flavonoid content of fermented oats. Similarly, Ðordevic et al. (2010) reported that the increase in total phenolic content and antioxidant activity through fermentation is significantly dependent on the species used. In the present study, the difference in biological activity between biomasses may be attributed to the variation in enzymes produced by microorganism species.

### Identification and quantification of volatile organic compounds

In the current study, six main groups of compounds acids, alcohols, aldehydes, phenols, esters, and ketones were identified in the whey powder, fungal biomass, and supernatant samples (Supplemental Table S1). The fermentation process has increased the diversity of acids in the biomass. This increase in acid diversity may result from the degradation of amino acids and the conversion of aldehydes by fungal enzymes, or from the breakdown of lipids, leading to the production of free fatty acids (Tian et al., 2023). It has also been reported that acidic compounds can be formed through the decomposition of esters (Qiu et al., 2023). Especially, both biomasses contained high concentrations of hexanoic acid (*A. oryzae* biomass, 355.21 mg/kg; *N. intermedia* biomass, 444.28 mg/kg).

1-octen-3-ol (often described as having a mushroom-like aroma) is one of the major volatile compounds produced by various fungal species such as *Aspergillus* spp., *Cladosporium* spp., *Fusarium* spp., and *Penicillium* spp (Iamanaka et al., 2014). In this study, 1-octen-3-ol was found in higher concentrations in *A. oryzae* biomass (129.35 µg/kg), *N. intermedia* biomass (128.06 µg/kg), and *N. intermedia* supernatant (43.67 µg/L) compared to whey powder (15.64 µg/kg). This compound is formed during fermentation as a result of the oxidative breakdown of linoleic acid by microbial lipoxygenases (Hwang and Kim, 2023; Wålinder et al., 2008). The biosynthesis of 1-octen-3-ol begins with the enzymatic cleavage of the double bonds at positions C-8 or C-10 in the linoleic acid backbone. Molecular oxygen was subsequently introduced at this site, leading to the formation of products 8-HPODE (hydroperoxyoctadecadienoic acid) and 10-HPODE. As a result, a portion of 10-HPODE is converted into 10-octadecenoic acid and 1-octen-3-ol (Kataoka et al., 2020; Wålinder et al., 2008). 1-octanol, which had earthy and nutty notes, was detected in all fermented products but was absent in the whey powder. The increase in alcohol groups with fermentation may be the result of the alcohol fermentation of lactose present in whey powder (Hwang and Kim, 2023).

1-pentanol (noted for its balsamic and fruity aroma), was specifically identified in the biomass of *A. oryzae* (82.55 µg/kg) and *N. intermedia* (82.86 µg/kg), but not in whey powder. Additionally, benzyl alcohol (characterized by sweet and floral notes) and phenylethyl alcohol (associated with honey, spice, and floral aromas) were found in *N. intermedia* biomass and supernatant, but not in whey powder or *A. oryzae* samples. Wickramasinghe and Munafo (2020) reported that benzyl alcohol was converted to benzyl aldehyde through the oxidation of aryl-alcohol oxidase, making benzyl alcohol the precursor of benzyl aldehyde. Phenylethyl alcohol is produced by fungi through both the oxidative deamination and de novo biosynthesis of phenylalanine (Martínez-Avila et al., 2018; Starzyńska-Janiszewska et al., 2024). Both, 1-pentanol and phenylethyl alcohol have been identified in various fermented foods, including *Neurospora*-type okara, broad bean koji, soy sauce (Peng et al., 2023; Qiu et al., 2023; Tian et al., 2023).

Hexanal (described as having grass, tallow, fatty notes), (Z)-2-heptanal (associated with soap, fat, almond aromas) and (E)-2-octenal (noted for green, nut, fatty characteristics) were detected exclusively in the biomass samples. Hexanal is known to form through the oxidation of polyunsaturated fatty acids such as linoleic acid (Hwang and Kim, 2023; Yoshizaki et al., 2010). The presence of these aldehydes only in the biomass suggests a correlation with the increasing linoleic acid content, which aligns with the observed rise in fat content within the biomasses. Notably, hexanal was also identified in amazake produced from stale bread using *A. oryzae* (Starzyńska-Janiszewska et al., 2024). Similar findings were observed by Qiu et al. (2023) in okara samples produced by *Neurospora crassa* fermentation.

When compared to whey powder, the intensities of nonanal and benzaldehyde increased in both biomass samples. Benzaldehyde has been reported to be synthesized from phenylalanine (Hwang and Kim, 2023; Starzyńska-Janiszewska et al., 2024)*. A. oryzae* biomass (88.69 µg/kg) had the highest benzaldehyde content compared to other samples. Similarly, the phenylalanine concentration was also the highest in *A. oryzae* biomass (3850.01 mg/kg). This finding aligns with previous research by Feng et al. (2013), which identified (E)-2-octenal and benzaldehyde as the most abundant compounds in the koji fermentation process using *A. oryzae*.

Phenols and ketones were not detected in the whey powder, but they were identified in the biomasses and supernatants. Within ketones, 3-octen-2-one and 2-undecanone were detected exclusively in biomass samples. Additionally, the ester compounds, methyl octanoate (orange) and methyl decanoate were also found solely in the biomass samples. This may be attributed to the oxidation and degradation of fatty acids or carotenoids that occur during fermentation (Cao et al., 2018).

### Sensory characteristics

**Figure 2.**
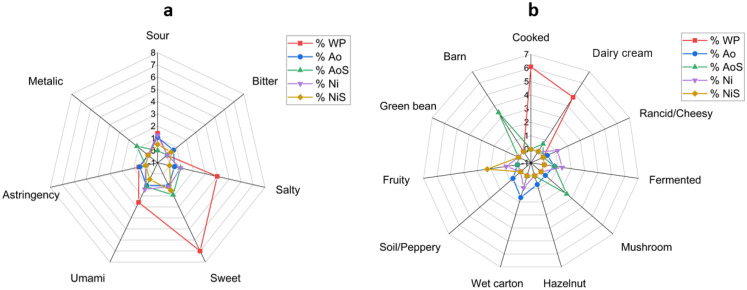
The descriptive taste (a) and flavor (b) properties of biomass and supernatant samples. Ao: dry biomass of *Aspergillus oryzae*; AoS: supernatant of *Aspergillus oryzae*; Ni: dry biomass of *Neusrospora intermedia*; NiS: supernatant of *Neurospora intermedia*; WP: whey powder.

Sensory characteristics of the samples were shown in Figure 2. It was determined that sweet taste decreased with fermentation when compared to whey powder, likely due to the fungi's utilization of lactose in the substrate. Glycine and alanine, known for their sweet taste contributions, may influence the perceived sweetness in the fermented products (Rohimah et al., 2021). Glutamic acid and aspartic acid are commonly associated with umami taste (Yay et al., 2024), explaining the presence of umami taste in the samples. Conversely, leucine, valine, and phenylalanine are linked to bitterness (Wikandari et al., 2021), and the higher concentration of these amino acids in the *A. oryzae* biomass may have contributed to the perception of bitter taste in this study. These findings were consistent with previous studies on oncom and tempeh prepared with *Rhizopus oligosporus*, which also detected sweet, bitter, and umami flavors (Rohimah et al., 2021; Utami et al., 2016).

The flavor profile of whey powder is typically characterized as cooked and dairy cream. However, fermentation with *A. oryzae* and *N. intermedia* significantly altered the sensory attributes of the biomasses and supernatants.

After fermentation with *A. oryzae*, rancid/cheesy notes were notably decreased, while new flavors emerged, including fermented, mushroom, hazelnut, wet carton, and soil/peppery. This aligns with findings from a previous study on wheat koji, where a nutty aroma increased throughout fermentation, as determined by descriptive sensory evaluation (Hwang and Kim, 2023). The supernatant of *A. oryzae* exhibited a flavor profile of creamy milk, fermented, mushroom, and barn, highlighting the complexity introduced through fermentation.

In contrast, fermentation with *N. intermedia* resulted in an increase in rancid and cheesy flavors within the biomass. However, it also contributed new sensory attributes such as fermented, wet carton, and fruity flavors. Notably, the *N. intermedia* supernatant had only a fruity flavor, suggesting a distinct flavor evolution compared to the *A. oryzae* supernatant. These changes in flavor profiles indicate the potential for enhancing the sensory characteristics of whey-based products through fungal fermentation.

### Principal component analysis applied to the obtained volatile organic compounds and descriptive sensory

**Figure 3.**
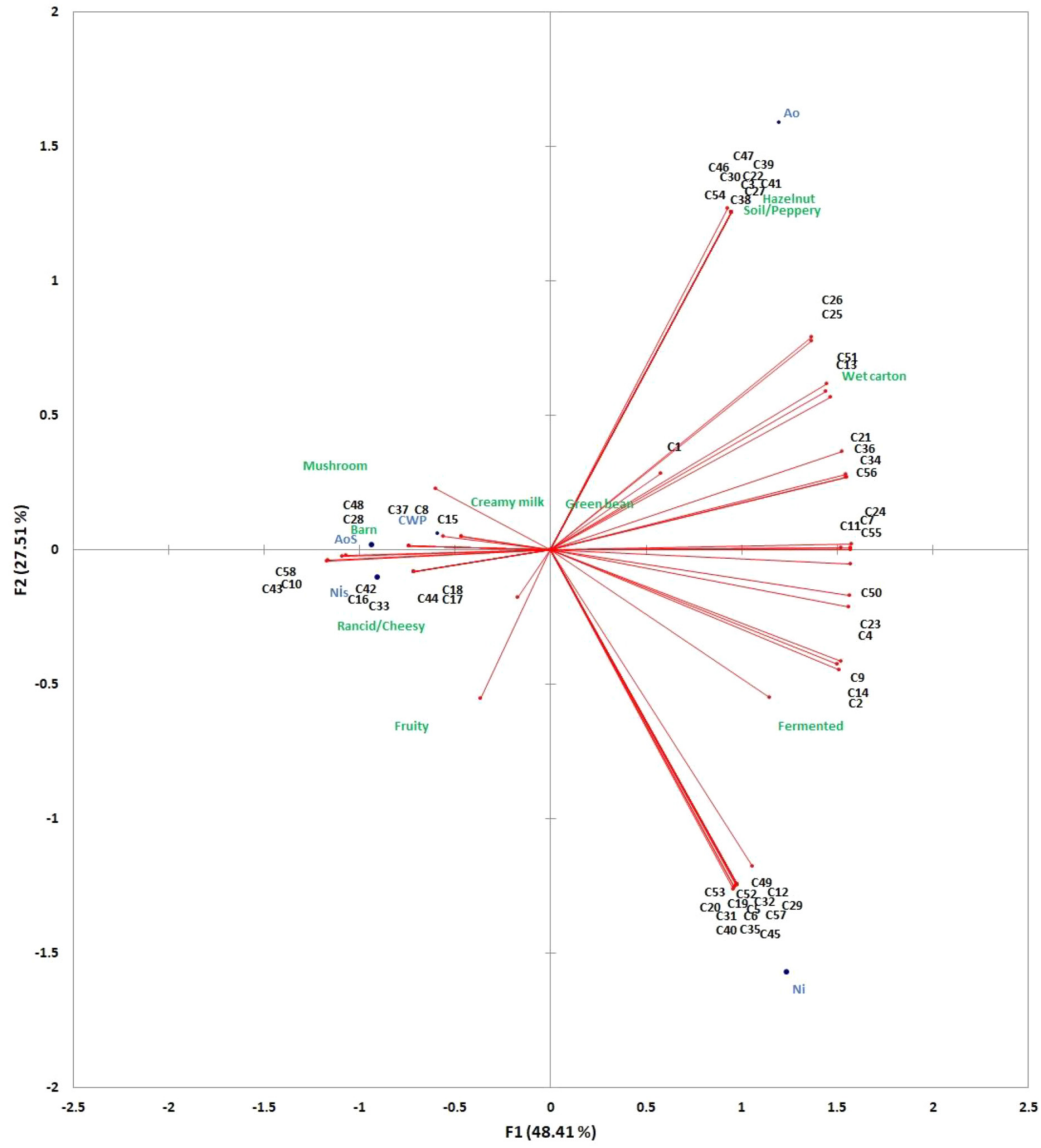
Relationship between volatile compounds and sensory properties of the whey powder, biomass, and supernatant samples. *The volatile compounds associated with the codes are specified in Supplemental Table S1.

Principal component analysis (PCA) analysis is a very useful tool in flavor science for revealing the relationship between volatile compounds and sensory properties of food samples. Based on the findings of PCA analysis, variations in fungal biomasses and whey powder regarding their volatile organic compounds (VOCs) profiles and sensory properties could be explained by two basic principal components. Both components were satisfactory to interpret variations with 76.92% of variance, with component PC1 48.41% and component PC2 27.51% (Figure 3).

Considering the PCA results, the fungal biomasses of *A. oryzae* and *N. intermedia* had different flavor characteristics compared with other samples. The aroma profile of *A. oryzae* biomass was well characterized by hazelnut and soil/peppery notes, which were found to be associated with esters such as methyl hexadecanoate, methyl tetradecanoate, methyl laurate, and pentanoic acid, as well as compounds like (E)-2-nonenal and 2-pentyl furan. In contrast, *N. intermedia* biomass was distinguished by a fermented flavor, with key contributors to its aroma profile including octanoic acid, methyl hexanoate, 3-methyl phenol, phenol, 6-methyl-5-hepten-2-one, phenethyl alcohol, and benzyl alcohol. It was found that *A. oryzae* supernatant and *N. intermedia* supernatant samples had similar characteristic sensory and aroma profiles and were well characterized with rancid/cheesy, barn aromas. 2-octanol, 3-octanone, 4-octanone and benzaothiazole were found to contribute the volatile profiles of both *A. oryzae* supernatant and *N. intermedia* supernatant. In the case of whey powder, 3-octane-2-ol and tridecyl 2-methoxyacetate were found to contribute to the volatile profile of whey powder, which were characterized dairy cream aroma.

## Conclusions

The fermentation of whey powder using *A. oryzae* and *N. intermedia* significantly enhances its nutritional value and sensory characteristics. For example, while *Aspergillus oryzae* stands out in terms of its mineral content, *N. intermedia* exhibits remarkable bioactivity properties. The key findings of this study are as follows:

- Nutritional Enhancement: The fungal biomass produced from fermentation exhibits a high crude protein content, which includes all essential amino acids, potentially meeting approximately 40% of an adult's daily protein requirements (per 100 g fungal biomass).
- Improved Lipid Content: Both fungal species increased the lipid content of the biomass compared to the whey powder, with notable levels of beneficial unsaturated fatty acids, including omega-6 and omega-9.
- Increased Bioactive Compounds: The fermentation process resulted in enhanced levels of total phenolic compounds, total flavonoids, and antioxidant activity in the fungal biomasses.
- Diverse Volatile Compounds and Sensory Profiles: Analysis of volatile compounds revealed that the characteristics of the biomass vary depending on the fungal species used for fermentation. This variability offers opportunities for creating distinct flavor profiles in the resulting products.

These findings provide valuable insights into the potential of using fungal fermentation to develop alternative food products. The enhanced nutritional profile and unique sensory attributes of the fermented whey biomass indicate promising applications in the food industry, paving the way for innovative products that cater to health-conscious consumers (Dean et al., 2015; Furey et al., 2022). While supernatants can be utilized as beverages, biomass can serve as a protein-enriching byproduct in various product formulations.

## Supplemental Material

sj-docx-1-fst-10.1177_10820132251368707 - Supplemental material for Enhancing nutritional value and flavor profiles of whey powder through fungal fermentation with *Aspergillus oryzae* and *Neurospora intermedia* Supplemental material, sj-docx-1-fst-10.1177_10820132251368707 for Enhancing nutritional value and flavor profiles of whey powder through fungal fermentation with *Aspergillus oryzae* and *Neurospora intermedia* by Burcu Kaya, Onur Güneşer, Mohammad J Taherzadeh, Yonca Karagül Yüceer and Taner Sar in Food Science and Technology International

sj-docx-2-fst-10.1177_10820132251368707 - Supplemental material for Enhancing nutritional value and flavor profiles of whey powder through fungal fermentation with *Aspergillus oryzae* and *Neurospora intermedia* Supplemental material, sj-docx-2-fst-10.1177_10820132251368707 for Enhancing nutritional value and flavor profiles of whey powder through fungal fermentation with *Aspergillus oryzae* and *Neurospora intermedia* by Burcu Kaya, Onur Güneşer, Mohammad J Taherzadeh, Yonca Karagül Yüceer and Taner Sar in Food Science and Technology International
