# Supplementary material for: Enhancing nutritional value and flavor profiles of whey powder through fungal fermentation with Aspergillus oryzae and Neurospora intermedia
Source: Food Sci Technol Int. 2025 Aug 17;32(6):724–37. doi: 10.1177/10820132251368707 (PMC13477504; doi:10.1177/10820132251368707)
Supplement: Supplementary file 2 [file sj-docx-2-fst-10.1177_10820132251368707.docx]

**Table S1.** Volatile compounds in whey powder, biomass and supernatant samples

| **No** | **Volatile compounds** | **RI** | **Aroma quality** | **Relative abundance of volatile compound (Mean±S.D)** | | | | |
| --- | --- | --- | --- | --- | --- | --- | --- | --- |
|  |  |  |  | **CWP (mg/kg)** | **Ao (mg/kg)** | **AoS (mg/L)** | **Ni (mg/kg)** | **NiS (mg/L)** |
|  | ***Acids*** |  |  |  |  |  |  |  |
| C1 | acetic acid | 1436 | sour | 261.91±34.25 | 263.39±10.57 | 264.21±78.43 | 222.27±13.98 | 3.53±0.27 |
| C2 | butanoic acid | 1599 | rancid, cheese, sweat | nd | 128.54±21.74 | nd | 207.12±33.69 | nd |
| C3 | pentanoic acid | 1703 | sweat | nd | 32.86±3.95 | nd | nd | nd |
| C4 | hexanoic acid | 1758 | sweat | nd | 355.21±73.28 | nd | 444.28±51.81 | nd |
| C5 | octanoic acid | 1927 | sweat, cheese | nd | nd | nd | 128.20±4.81 | nd |
|  | ***Alcohols*** |  |  | **CWP (µg/kg)** | **Ao (µg/kg)** | **AoS (µg/L)** | **Ni (µg/kg)** | **NiS (µg/L)** |
| C6 | 3-methyl-2-pentanol | 1189 |  | nd | nd | nd | 101.08±33.70 | 3.56±0.14 |
| C7 | 1-pentanol | 1238 | balsamic,fruit | nd | 82.55±9.42 | nd | 82.86±32.77 | nd |
| C8 | 3-octane-2-ol | 1329 |  | 23.85±4.46 | nd | nd | nd | nd |
| C9 | 1-hexanol | 1337 | resin, flower, green | nd | 43.91±6.82 | nd | 68.07±24.24 | 0.36±0.04 |
| C10 | 3-octanol | 1377 | moss, nut, mushroom | nd | nd | 0.57±0.11 | nd | 0.25±0.05 |
| C11 | 1-octen-3-ol | 1431 | mushroom | 15.64±5.38 | 129.35±26.57 | 4.28±0.40 | 128.06±41.86 | 43.67±3.70 |
| C12 | 6-methyl-5-hepten-2-ol, | 1443 |  | nd | 17.42±3.15 | nd | 265.20±79.46 | nd |
| C13 | 2-ethyl hexanol | 1466 | rose, green | 29.05±12.64 | 89.20±7.92 | 1.40±0.03 | 52.01±13.29 | 1.27±0.17 |
| C14 | 1-octanol | 1529 | moss, nut, mushroom | nd | 48.08±0.31 | 6.22±0.59 | 74.41±22.79 | 0.88±0.06 |
| C15 | (E)-2-octen-1-ol, | 1586 | soap, plastic | nd | nd | 2.01±0.29 | nd | nd |
| C16 | 1-nonanol | 1627 | fat, green | nd | nd | nd | nd | 0.28±0.03 |
| C17 | 2-furanmethanol | 1633 | burnt | nd | nd | nd | nd | 0.27±0.01 |
| C18 | 1-dodecanol | 1714 | fat, wax | nd | nd | nd | nd | 0.50±0.06 |
| C19 | benzyl alcohol | 1779 | sweet, flower | nd | nd | nd | 24.49±8.49 | 0.32±0.02 |
| C20 | phenylethyl alcohol | 1795 | honey, spice, rose, lilac | nd | nd | nd | 14.94±0.47 | 0.70±0.08 |
|  | ***Aldehydes*** |  |  |  |  |  |  |  |
| C21 | hexanal | 1088 | grass, tallow,fat | nd | 906.86±140.66 | nd | 632.96±191.40 | nd |
| C22 | octanal | 1283 | fat, soap, lemon, green | nd | 27.28±0.66 | 1.17±0.09 | nd | nd |
| C23 | (Z)-2-heptenal | 1321 | soap, fat, almond | nd | 49.58±1.93 | nd | 59.40±10.42 | nd |
| C24 | nonanal | 1389 | fat, citrus, green | 23.36±3.57 | 83.08±17.67 | 0.88±0.10 | 83.13±29.96 | 1.28±0.17 |
| C25 | (E)-2-octenal | 1424 | green, nut, fat | nd | 55.55±19.70 | nd | 22.17±6.13 | nd |
| C26 | benzaldehyde | 1515 | almond, burnt sugar | 16.76±7.58 | 88.69±3.05 | 0.38±0.02 | 37.89±18.49 | 0.56±0.17 |
| C27 | (E)-2-nonenal | 1521 | cucumber, fat, green | nd | 36.09±3.59 | nd | nd | nd |
| C28 | (E,E)-2,4-octadienal | 1571 | green, seaweed, cucumber | nd | nd | 0.54±0.20 | nd | nd |
| C29 | (E)-2-decenal | 1625 |  | nd | nd | nd | 20.05±1.87 | nd |
|  | ***Phenols*** |  |  |  |  |  |  |  |
| C30 | 2-methyl phenol | 1877 | phenol | nd | 14.61±0.22 | nd | nd | nd |
| C31 | phenol | 1881 | phenol | nd | nd | nd | 28.66±8.24 | nd |
|  | ***Esters*** |  |  |  |  |  |  |  |
| C32 | methyl hexanoate | 1182 | sweat | nd | nd | nd | 77.06±34.02 | nd |
| C33 | butyl butyrate ester | 1210 |  | nd | nd | nd | nd | 0.23±0.04 |
| C34 | methyl octanoate | 1380 | orange | nd | 20.14±3.60 | nd | 15.41±5.39 | nd |
| C35 | 3-hydroxymandelic acid, ethyl ester, di-TMS | 1483 |  | nd | nd | nd | 46.33±16.43 | 1.50±0.12 |
| C36 | methyl decanoate | 1571 | wine | nd | 17.67±3.22 | nd | 13.63±6.58 | nd |
| C37 | tridecyl 2-methoxyacetate | 1665 |  | 9.76±3.46 | nd | nd | nd | nd |
| C38 | methyl laurate | 1738 | fat, coconut | nd | 21.20±5.49 | nd | nd | nd |
| C39 | methyl tetradecanoate | 1881 | orris | nd | 32.76±1.47 | nd | nd | nd |
| C40 | Isopropyl myristate | 1905 |  | nd | nd | nd | 53.76±15.53 | nd |
| C41 | Methyl hexadecanoate | 2042 |  | nd | 34.11±6.73 | nd | nd | nd |
|  | ***Ketones*** |  |  |  |  |  |  |  |
| C42 | 4-octanone | 1219 |  | nd | nd | 0.52±0.11 | nd | 0.38±0.03 |
| C43 | 3-octanone | 1246 | herb, butter, resin | nd | nd | 1.68±0.12 | nd | 1.19±0.22 |
| C44 | 2,6-dimethyl-3-hexanone, | 1285 |  | nd | nd | nd | nd | 1.07±0.22 |
| C45 | 2-octen-4-one | 1286 |  | nd | nd | nd | 147.49±28.16 | nd |
| C46 | 1-octen-3-one | 1294 | mushroom, metal | nd | 80.72±17.41 | nd | nd | nd |
| C47 | 1-hydroxy-3-methyl-2-butanone | 1329 |  | nd | 31.27±4.76 | nd | nd | nd |
| C48 | 1-hydroxy-2-pentanone | 1330 |  | nd | nd | 1.32±0.19 | nd | nd |
| C49 | 6-methyl-5-hepten-2-one, | 1331 |  | nd | nd | nd | 127.40±31.73 | nd |
| C50 | 3-octen-2-one | 1403 | nut, crushed bug | nd | 31.25±2.70 | nd | 33.30±12.72 | nd |
| C51 | 2-undecanone | 1577 | orange, fresh, green | nd | 34.76±5.10 | nd | 17.86±8.15 | nd |
| C52 | (E)- 6,10-dimethyl-5,9-undecadien-2-one, -, | 1768 |  | nd | nd | nd | 39.87±15.16 | nd |
| C53 | 3-buten-2-one, 4-(2,6,6-trimethyl-1-cyclohexen-1-yl)-, (E)- | 1826 |  | nd | nd | nd | 46.41±12.83 | nd |
|  | ***Miscellaneous*** |  |  |  |  |  |  |  |
| C54 | 2-pentylfuran | 1227 | green bean, butter | nd | 21.35±3.62 | nd | nd | nd |
| C55 | 1,3-Hexadiene, 3-ethyl-2-methyl- | 1415 |  | nd | 12.69±3.60 | nd | 12.84±7.29 | nd |
| C56 | methoxy-phenyl oxime | 1707 |  | 77.43±28.52 | 287.29±74.58 | 7.98±1.03 | 230.04±78.27 | 4.68±0.32 |
| C57 | (Z)- 7,11-dimethyl-3-methylene-1,6,10-dodecatriene, | 1638 |  | nd | nd | nd | 50.23±18.05 | 0.51±0.17 |
| C58 | benzothiazole | 1852 | gasoline, rubber | nd | nd | 0.97±0.13 | nd | 0.47±0.03 |

*nd:Not detected, SE: standard error.
